# Prevalence, Distribution and Antimicrobial Susceptibility of *Enterobacteriaceae* and Non‐Fermenting Gram‐Negative Bacilli Isolated From Environmental Samples in a Veterinary Clinical Hospital in Madrid, Spain

**DOI:** 10.1111/1758-2229.70055

**Published:** 2024-12-23

**Authors:** Jesús Antonio Pérez Jiménez, Silvia Penelo Hidalgo, María‐Rosario Baquero Artigao, Gustavo Ortiz‐Díez, Tania Ayllón Santiago

**Affiliations:** ^1^ Facultad de Ciencias de la Salud Universidad Alfonso X el Sabio Madrid Spain; ^2^ Servicio de Urgencias, Hospitalización y UCI, Hospital Clínico Veterinario Complutense Universidad Complutense de Madrid Madrid Spain; ^3^ Hospital Clínico Veterinario Complutense Universidad Complutense de Madrid Madrid Spain; ^4^ Departamento de Genética, Fisiología y Microbiología, Facultad de Ciencias Biológicas Universidad Complutense Madrid Spain

**Keywords:** antimicrobial resistance, *Enterobacteriaceae*, non‐fermenting gram‐negative bacilli, nosocomial infections, veterinary hospital

## Abstract

Managing infections caused by multidrug‐resistant Gram‐negative bacilli is a major public health concern, particularly in hospitals where surfaces can act as reservoirs for resistant microorganisms. Identifying these bacteria in hospital environments is crucial for improving healthcare safety. This study aimed to analyse environmental samples from a veterinary hospital to identify prevalent microorganisms and detect antimicrobial resistance patterns. A total of 183 surface samples were collected from 26 areas at the Veterinary Clinical Hospital of Alfonso X el Sabio University in Madrid. The isolated strains were identified, and susceptibility profiles were determined via the disk diffusion method. Clonality analysis was performed using pulsed‐field gel electrophoresis. In total, 109 strains were isolated: 76.15% from the *Enterobacteriaceae* family and 23.85% non‐fermenting Gram‐negative bacilli. The isolates included *Klebsiella, Enterobacter, Escherichia* and *Pseudomonas* species, which could include high‐risk clones, given their ability to carry several antimicrobial resistance genes. The equine area had the highest number of isolates (*n* = 71), accounting for 65% of the total. High resistance indices were observed against at least five of the 16 antibiotics tested, indicating significant multidrug resistance. Clonality analysis suggested potential cross‐transmission within the facility. This study sampled hospital surfaces but not personnel or animals, making contamination sources unclear. Without resampling, the effectiveness of cleaning protocols remains uncertain. Results suggest that hospital staff play a key role in bacterial transmission. The lack of specialised preventive measures in veterinary hospitals highlights a need for further research and improvement.

## Graphical abstract

This study examined bacterial contamination on surfaces within a veterinary hospital in Madrid, isolating 109 bacterial strains, predominantly Enterobacteriaceae and non‐fermenting Gram‐negative bacilli. The highest contamination was found in the equine area, with strains showing resistance to multiple antibiotics. These findings highlight the potential for cross‐transmission within the facility and the need for improved infection control protocols in veterinary environments.

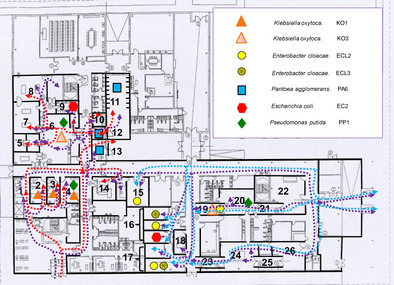

## 1 Introduction

Antimicrobial resistance (AMR) is a major public health concern (Frieri, Kumar, and Boutin 2017; Laxminarayan et al. 2013; World Health Organization 2022), for which surveillance plans have been developed to monitor the appropriate use of the implicated drugs (European Parliament 2023; European Commission 2017; Smith et al. 2016; Schwarz, Kehrenberg, and Walsh 2001). In veterinary medicine, the indiscriminate and inappropriate use of antibiotics in animals has led to the development of resistance – whose mechanisms have been investigated – in pathogens that affect both animals and humans (Wu 2019; Iwu, Korsten, and Okoh 2020; Smet et al. 2011; Hammerum and Heuer 2009; Barza 2002). Most risk factors for the development of nosocomial infections described in human medicine can also be applied to veterinary medicine (Kisani et al. 2016; Milton 2015; Mocherniuk et al. 2022). Multidrug‐resistant (MDR) Gram‐negative bacilli, including extended‐spectrum beta‐lactamase (ESBL), carbapenemase‐producing *Enterobacteriaceae*, fluoroquinolone‐resistant *Pseudomonas aeruginosa* , carbapenemase‐producing *P. aeruginosa* and *Acinetobacter baumannii* , have developed complex resistance mechanisms and have become pandrug‐resistant with the potential for horizontal gene transfer through mobile genetic elements (van Hoek et al. 2011; Sultan et al. 2018; Mancuso et al. 2021; Asenjo, Oteo‐Iglesias, and Alós 2021; Wilson and Török 2018). These bacteria can cause nosocomial infections as well as infections in non‐hospitalised patients (Milton 2015; Köck et al. 2017).

Environmental contamination is a major contributor to nosocomial infections in veterinary hospitals. Surfaces are effective vehicles for transmission and serve as reservoirs for different microorganisms, including multidrug‐resistant bacteria, making them important components of hospital environmental monitoring programs (Otter, Yezli, and French 2011; Assadian et al. 2021; Alfa et al. 2015; Simmonds‐Cavanagh 2022).

This study aimed to characterise the bacterial populations in environmental samples obtained from the Veterinary Clinical Hospital of Alfonso X el Sabio University (HCV‐UAX), particularly Gram‐negative bacilli. The isolated bacterial strains were analysed to determine their sensitivity and/or resistance profiles to the most commonly used antibiotics in veterinary medicine. Finally, the clonal relationships among different isolates of the same species were characterised using pulsed‐field gel electrophoresis (PFGE) to determine bacterial spreading.

## 2 Materials and Methods

### 2.1 Study Area and Sample Collection and Isolation

**FIGURE 1:**
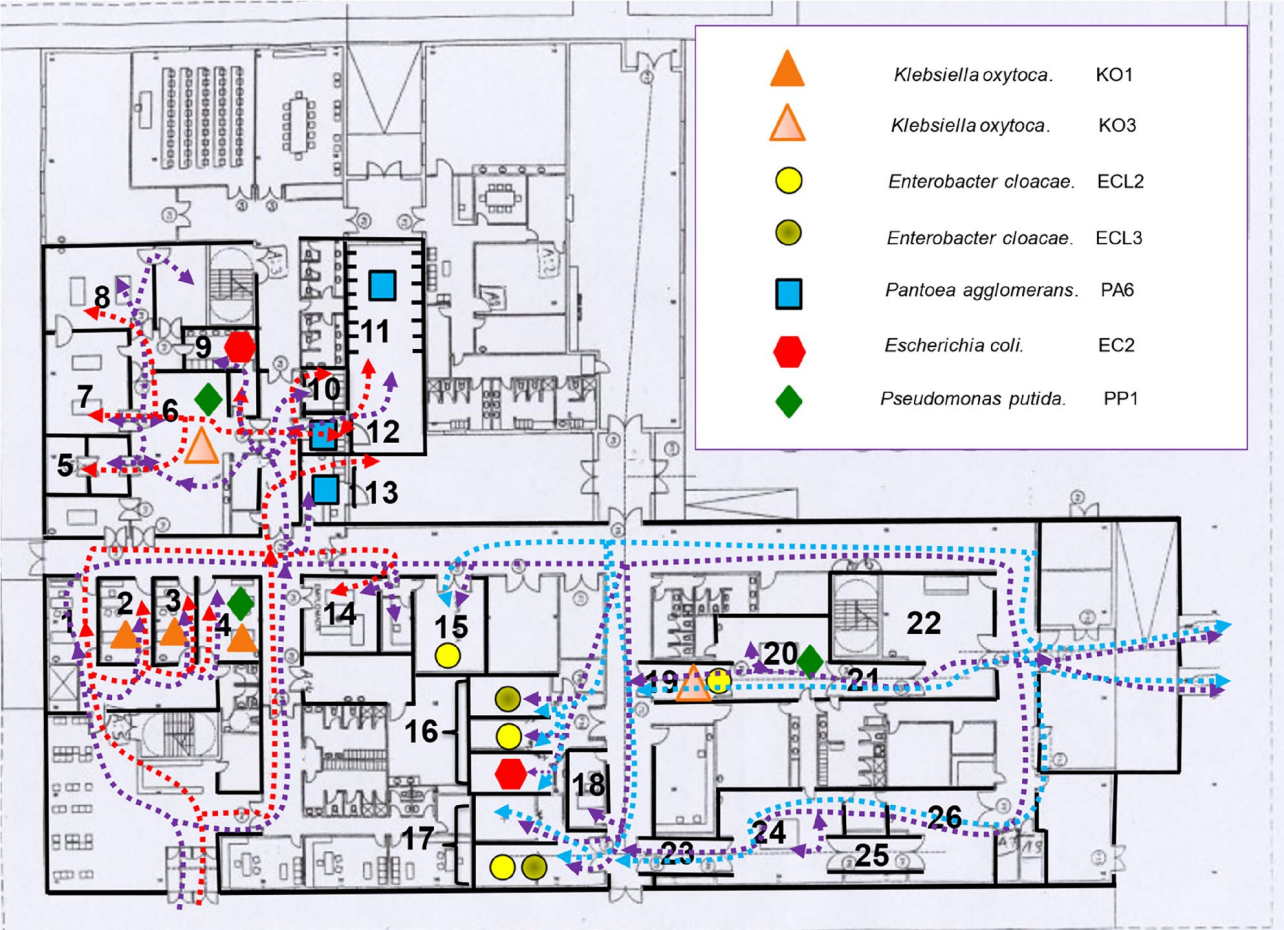
Map showing the layout of sampling rooms at the Veterinary Clinical Hospital of Alfonso X el Sabio University (HCV‐UAX), illustrating the movement flow of small animals, horses and personnel, along with the clones identified in the study. Red arrows indicate small animal (dog) transit, blue arrows show horse transit and purple arrows represent personnel movement. 1. Consultation room 1; 2. Consultation room 2; 3. Consultation room 3; 4. Consultation room 4; 5. Small animal recovery area; 6. ICU Hospitalisation; 7. Small animal operating room 1; 8. Small animal operating room 2; 9. Small animal anaesthesia induction room; 10. Infectious Small Animal Hospitalisation; 11. Large dog hospitalisation room; 12. Small dog hospitalisation room; 13. Cats hospitalisation room; 14. X‐Ray and CT room for small animals; 15. X‐Ray room for equines; 16. Equine stables ICU‐1 (3 cages); 17. Equine stables ICU‐2 (2 cages); 18. Residents area; 19. Equine recovery room 1; 20. Equine operating room 1; 21. Equine anaesthesia induction room 1; 22. Equine examination room 1; 23. Equine recovery room 2; 24. Equine operating room 2; 25. Equine anaesthesia induction room 2; 26. Equine examination room.

This descriptive, cross‐sectional, observational study was conducted using a convenience sample from March to April 2016. A total of 183 samples were collected from different areas of HCV‐UAX using extensive environmental sampling. The HCV‐UAX Veterinary Clinical Hospital is divided into two primary sections: one for small animals and another for large animals (equines), encompassing a total of 26 different areas. The small animal section includes four consultation rooms, two operating rooms, one recovery area, a hospitalisation room for large dogs, a hospitalisation room for small dogs, a hospitalisation room for cats and a specific area for the hospitalisation of infectious small animals. For anaesthesiology, the small animal section features an anaesthesia induction room. Diagnostic imaging is facilitated by a combined X‐ray and CT room. The large animal section includes two equine examination rooms, two operating rooms and two intensive care units. Additionally, there is an X‐ray room for equines. Anaesthesiology and recovery facilities for large animals include two equine anaesthesia induction rooms and two recovery rooms. Additional facilities include a resident area (Figure 1).

Different surfaces were selected for sampling in each area based on their operational and functional characteristics, including computer keyboards, worktables, sink tables, auxiliary tables, stretchers, countertop instrument cabinets, doors, cages (with or without animals inside) and different types of cabinets, refrigerators, walls, floors, horse stalls, scales and carts.

The samples were collected using sterile cotton swabs moistened with sterile distilled water. The sampling process comprised rotating and moving the swab horizontally from the inside to the outside of a 10 cm^2^ area for 10 s at each site. Samples were immediately cultured on McConkey agar (OXOID Ltd., Basingstoke, UK) and incubated at 37°C. McConkey agar was selected for this study due to its efficacy in isolating Gram‐negative bacteria (Allen 2016), which are the primary focus of our research. We ensured that colonies with different morphologies were analysed to capture the diversity of bacterial species present in the samples. Bacterial growth was observed 24, 48 and 72 h after culture. Isolates showing positive growth were re‐isolated in the same medium and incubated again at 37°C for 24 h to obtain pure cultures of all strains from each sample.

### 2.2 Bacterial Identification

The isolated strains were identified using different techniques (Isenberg 2004; Bou et al. 2011; Fernández et al. 2010), including biochemical methods such as the analytical profile index (API 20E; BioMérieux, Madrid, Spain), proteomic techniques such as matrix‐assisted laser desorption/ionisation‐time of flight (MALDI‐TOF mass spectrometry; Bruker Daltonics, Bremen, Germany) and PCR for 16S gene amplification at the Microbiology Service Laboratories of the Ramón y Cajal University Hospital in Madrid. The primer pair used for molecular identification included 16SF: 5´‐AGAGTTTGATCATGGCTCAG‐3′ (Forward) and 16SR: 5´‐CGGTTACCTTGTTACGACTT‐3′ (Reverse). PCR products were purified using an ExoSAP‐IT purification kit (Thermo Fisher Scientific, Waltham, MA, USA), and automated sequencing was performed by Macrogen (Seoul, Korea) using an ABI Prism 377 Automated Sequencer (Applied Biosystems, Foster City, CA, USA). The obtained sequences were subjected to bioinformatics analysis using Chromas (version 2.32; Technelysium Pty. Ltd., South Brisbane, QLD, Australia) and sequence comparison and alignment were performed using the BLAST programme (www.ncbi.nlm.nih.gov).

### 2.3 Antibiotic Susceptibility Profile

The disk diffusion method was used to determine the susceptibility profiles of the isolated strains, wherein the size of the inhibition zone was related to the minimum inhibitory concentration obtained using the dilution method (King and Brown 2001; Andrews 2001). The antibiotics selected included amoxicillin, amoxicillin‐clavulanic acid, aztreonam, cefoxitin, cefotaxime, ceftazidime, imipenem, meropenem, gentamicin, amikacin, nalidixic acid, ciprofloxacin, trimethoprim‐sulfamethoxazole, colistin, tetracycline and tigecycline. The concentration in each disk was the same for all antibiotics and was equal to 30 μg. The experimental procedure was conducted according to protocols described in literature (*Enfermedades Infecciosas y Microbiología Clínica* 2013). Clinical and Laboratory Standards Institute (CLSI) criteria (Clinical and Laboratory Standards Institute. CLSI 2015; Clinical and Laboratory Standards Institute (CLSI) 2015; Clinical and Laboratory Standards Institute (CLSI) 2012) were used to interpret the results.

### 2.4 Clonality Study

Clonality analysis of all identified bacterial species was performed using PFGE with a CHEF DR‐III apparatus (Bio‐Rad Laboratories, Hercules, CA, USA). The experimental procedure consisted of several stages: (i) in situ DNA extraction from agarose blocks using the PulseNet protocol (CDC, Atlanta, GA, USA) (The National Molecular Subtyping Network for Foodborne Disease Surveillance 2005), with specific steps for *Enterobacteriaceae* and *Pseudomonas* spp., and (ii) digestion of the extracted DNA using the restriction enzymes XbaI and SpeI for *Enterobacteriaceae* and *Pseudomonas* spp., respectively.

### 2.5 Statistical Analysis

Categorical variables, including the number of isolates, sex, species, API test results, MALDI‐TOF mass spectrometry, PCR results, bacterial susceptibility, number of clones and pulsotype, were represented as frequency distributions along with their corresponding percentages.

## 3 Results

### 3.1 Bacterial Isolation

Of the 183 samples collected for the survey, 109 (59.56%) were classified as Gram‐negative, lactose‐fermenting, or non‐fermenting bacteria based on their behaviour on MacConkey Agar culture medium. The highest number of isolates was found in the equine area, specifically in intensive care unit (ICU)‐1 and ICU‐2 stalls (Figure S1), followed by residential areas. Table 1 shows the remaining sample collection results according to the area studied at HCV‐UAX.

**TABLE 1:** Number of isolates and their isolation area at the Alfonso X el Sabio Veterinary Clinical Hospital.

Location	Total number of isolates (*n*)	Number of isolates by sampling zone (*n*)
Consultation Room 2	3	Computer keyboard (1), work table (1), instrument display case (1)
Consultation Room 3	1	Work table (1)
Consultation Room 4	5	Computer keyboard (2), work Table 2 (1), ultrasound scanner (1), countertop (1)
ICU Hospitalisation	6	Cage 4 (1), cage 7 (1), cage 8 (1), cage 11 (1), cage 15 (1), stretcher 3 (1)
Infectious small animal hospitalisation	1	Cage 3 (1)
Cat Hospitalisation room	5	Cage 2 (2), cage 7 (2), countertop (1)
Small dog hospitalisation room	3	Worktop 1 (1), worktop 2 (2)
Large Dog Hospitalisation room	10	Cage 4 (1), cage 9 (1), cage 12 (2), cage 13 (1), cage 14 (1), cage 15 (1), table (2), worktop (1)
Small animal Anaesthesia Induction Room	1	Table (1)
Small animal Recovery area	1	Cage 3 (1)
X‐ray and CT room for small animals	2	X‐ray Stretcher (1), countertop (1)
X‐ray room for equines	3	Table (3)
Equine stables ICU‐1 (3 cages)	21	Wall 1 (2), Gate 1 (2), stable 2/floor (5), wall 2 (6), gate 2 (3), stable 3/floor (1), wall 3 (2)
Residents area	9	Worktop 1 (1), work table (3), cupboard (2), table (1), door (2)
Equine stables ICU‐2 (2 cages)	16	Stable 1/floor (4), door 1 (4), stable 2 /floor (1), wall 2 (3), countertop (2), floor above (2)
Equine Examination Room 1	2	Floor (2)
Equine Anaesthesia Induction Room 1	4	Floor (4)
Equine Examination Room 2	6	Colt (3), cabinet (1), floor (2)
Equine Recovery Room 1	4	Floor (2), door (2)
Equine Recovery Room 2	3	Floor (1), door (2)
Equine Operating Room 1	1	Stretcher (1)
Equine Operating Room 2	2	Stretcher (1)

Abbreviations: CT, computed tomography; ICU, intensive care unit.

### 3.2 Bacterial Identification

Of the 109 isolated samples, 83 (76.15%) were identified as *Enterobacteriaceae* and 26 (23.85%) as non‐fermenting gram‐negative bacteria (NFGNB). The most frequently identified species of *Enterobacteriaceae* were *Enterobacter cloacae* (28.91%), *Klebsiella oxytoca* (11.66%) and *Escherichia coli* (13.25%). Regarding NFGNB, the most frequently identified species were *Pseudomonas* spp. (26.92%), *Pseudomonas putida* , *Pseudomonas stutzen* (19.23% each) and *Pseudomonas orzihabitants* (15.38%). Other *Enterobacteriaceae* and NFGNB species identified are shown in Table 2.

**TABLE 2:** Identification of isolated bacteria, number of isolates, and percentage from the total isolated.

	Species		Number of isolates (*n*)	Percentage (*n*/*N*) %
*Enterobacteriaceae*	*Klebsiella oxytoca*		13	15.66
	*Klebsiella pneumoniae*		1	1.21
	*Enterobacter* spp.		3	3.61
	*Enterobacter cloacae*		24	28.91
	*Enterobacter cobei*		1	1.21
	*Pantoea* spp.		5	6.02
	*Pantoea agglomerans*		9	10.84
	*Pantoea conspicua*		1	1.21
	*Pantoea vagans*		1	1.21
	*Pantoea eucrina*		1	1.21
	*Leclercia adecarboxylata*		5	6.02
	*Escherichia coli*		11	13.25
	*Escherichia vulneris*		1	1.21
	*Raoultella* spp.		1	1.21
	*Raoultella ornithinolytica*		2	2.41
	*Raoultella terricola*		3	3.61
	*Citrobacter freundii*		1	1.21
Total *Enterobacteriaceae* (*N* = 83)				100%
NFGNB	*Pseudomonas* spp.		7	26.92
	*Pseudomonas putida*		5	19.23
	*Pseudomonas orzyhabitans*		4	15.38
	*Pseudomonas mendocina*		2	7.69
	*Pseudomonas stutzeri*		5	19.23
	*Pseudomonas fluorescens*		1	3.85
	*Pseudomonas alcaliphila*		1	3.85
	*Stenotrophomonas maltophila*		1	3.85
Total NFGNB (*N* = 26)				100%

Abbreviation: NFGNB, non‐fermenting Gram‐negative bacteria.

### 3.3 Antimicrobial Susceptibility

Nineteen (22.89%) of the 83 enterobacterial strains isolated and 7 (26.92%) of the 26 NFGNB isolates were sensitive to all antibiotics tested, as shown in Table 3.

**TABLE 3:** Bacterial species susceptible to all antibiotics tested.

	Species	Percentage (%)
*Enterobacteriaceae*	*Enterobacter cloacae*	4.16
	*Pantoea* spp.	40.00
	*Pantoea agglomerans*	88.88
	*Pantoea vagans*	100
	*Pantoea eucrina*	100
	*Escherichia coli*	45.45
	*Citrobacter freundii*	100
NFGNB	*Pseudomonas oryzihabitans*	50.00
	*Pseudomonas mendocina*	50.00
	*Pseudomonas* spp.	42.85
	*Pseudomonas fluorescens*	100

Abbreviation: NFGNB, non‐fermenting Gram‐negative bacteria.

Both *Enterobacteriaceae* and NFGNB showed the highest resistance to amoxicillin, followed by trimethoprim‐sulfamethoxazole. *Enterobacteriaceae* were most susceptible to imipenem and colistin, followed by tigecycline and meropenem, while NFGNB were most susceptible to colistin and tigecycline, followed by tetracycline, amikacin, nalidixic acid, imipenem and ceftriaxone. Table 4 shows the resistance levels of the isolated species to the investigated antibiotics. The results are based on the number of strains isolated from each species.

**TABLE 4:** Global antimicrobial susceptibility profile.

Antibiotic	*Enterobacteriaceae*			NFGNB		
	Resistant (%)	Intermediate (%)	Sensible (%)	Resistant (%)	Intermediate (%)	Sensible (%)
AMX	71.08	1.2	27.72	46.15	0.00	53.85
AMC	32.53	10.84	56.63	15.38	0.00	84.62
ATM	14.45	6.02	79.53	15.38	69.23	15.39
FOX	31.32	9.63	59.05	23.07	0.00	76.93
CTX	27.71	14.45	57.84	3.84	0.00	96.16
CAZ	26.5	1.20	72.3	7.69	0.00	92.31
IPM	0.00	0.00	100	3.84	0.00	96.16
MEM	3.61	0.00	96.39	3.84	0.00	96.16
GM	50.6	1.20	48.2	23.07	0.00	76.93
AN	6.02	1.20	92.78	0.00	3.84	96.16
NA	14.45	4.81	80.74	3.84	0.00	96.16
CIP	6.02	2.40	91.58	7.69	0.00	91.31
SXT	56.02	1.20	42.18	42.3	0.00	57.70
CS	0.00	0.00	100	0.00	0.00	100
TE	51.8	0.00	48.2	3.84	0.00	96.16
TGC	1.20	1.20	97.6	0.00	0.00	100

Abbreviations: AMC, amoxicillin‐clavulanic acid; AMX, amoxicillin; AN, amikacin; ATM, aztreonam; CAZ, ceftazidime; CIP, ciprofloxacin; CS, colistin; CTX, cefotaxime; FOX, cefoxitin; GM, gentamicin; IPM, imipenem; MEM, meropenem; NA, nalidixic acid; NFGNB, non‐fermenting Gram‐negative bacteria; SXT, trimethoprim/sulfamethoxazole; TE, tetracycline; TGC, tigecycline.

### 3.4 Clonality

After PFGE, the phylogenetic relationships of the strains isolated from the different sites were determined. The highest number of clones was obtained from *E. cloacae* , with 12 different clones or pulse types identified, followed by *K. oxytoca* with 7 different clones and *Pantoea agglomerans* and *E. coli* with 6 different pulse types each. The results are summarised in Table 5.

**TABLE 5:** Number of clones by isolate and species identified.

	Species	Number of isolates (*n*)	Number of clones or pulse types (*n*)
*Enterobacteriaceae*	*Klebsiella oxytoca*	13	7
	*Klebsiella pneumoniae*	1	1
	*Enterobacter* spp	3	3
	*Enterobacter cloacae*	24	12
	*Enterobacter kobei*	1	1
	*Pantoea* spp	5	3
	*Pantoea agglomerans*	9	6
	*Pantoea conspicua*	1	1
	*Pantoea vagans*	1	1
	*Pantoea eucrina*	1	1
	*Leclercia adecarboxylata*	5	5
	*Escherichia coli*	11	6
	*Escherichia vulneris*	1	1
	*Raoultella* spp	1	1
	*Raoultella ornithinolytica*	2	1
	*Raoultella terrigena*	3	2
	*Citrobacter freundii*	1	1
NFGNB	*Pseudomonas* spp	7	7
	*Pseudomonas putida*	5	2
	*Pseudomonas orzyhabitans*	4	2
	*Pseudomonas mendocina*	2	1
	*Pseudomonas stutzeri*	5	4
	*Pseudomonas fluorescens*	1	1
	*Pseudomonas alcaliphila*	1	1
	*Stenotrophomonas maltophilia*	1	ND*

Abbreviations: ND*, not determined by PFGE; NFGNB, non‐fermenting Gram‐negative bacteria.

### 3.5 Bacterial Spreading

PFGE analysis allowed the construction of a map of the locations of clone distribution across different sampling areas (Figure 1), providing an overview of bacterial dispersion. This type of approach allows us to improve our understanding of the spreading of antibiotic‐resistant bacteria in our environment. As shown in Figure 1, there is no direct contact between small animals (red arrows) and equines (blue arrows). However, personnel (purple arrows) move between both areas. Several identical clones were identified in both hospital sections, indicating the spreading of the same bacterial strains across distinct areas. The different isolated clones and their locations are listed in Table 6.

**TABLE 6:** Location of isolated clones at the Alfonso X el Sabio Veterinary Clinical Hospital.

Species	Pulse‐type (number of isolates)	Localisation (number of clones)	Resistance profile
*Klebsiella oxytoca*	KO1 (5)	Consultation 2 (1) Consultation 3 (1) Consultation 4 (3)	AMX
*Klebsiella oxytoca*	KO2	ICU Hospitalisation	TE
*Klebsiella oxytoca*	KO3 (3)	ICU Hospitalisation (1), Equine Recovery Room 1 (2)	AMX, AMC, FOX, GM, NA, CIP, SXT, TE
*Klebsiella oxytoca*	KO4	Equine stables ICU‐2 (2 cages)	AMX
*Klebsiella oxytoca*	KO5	Consultation 4	AMX, AMC, FOX, MEM, SXT, TGC
*Klebsiella oxytoca*	KO6	Equine stables ICU‐1 (3 cages)	AMX, GM, SXT, TE
*Klebsiella oxytoca*	KO7	Residents' area	AMX, SXT
*Klebsiella pneumoniae*	KP1	Equine stables ICU‐2 (2 cages)	AMX, FOX, CTX, GM, SXT, TE
*Enterobacter cloacae*	ECL1 (2)	Equine stables ICU‐1 (3 cages) (3)	AMX, AMC, ATM, FOX, CTX, CAZ, GM, SXT, TE
*Enterobacter cloacae*	ECL2 (5)	X‐Ray room for equines (1), Equine stables ICU‐2 (2 cages) (2), Equine Recovery Room 1 (1) Equine stables ICU‐1 (3 cages) (1)	AMX, AMC, ATM, FOX, CTX, CAZ, GM, SXT, TE
*Enterobacter cloacae*	ECL3 (3)	Equine stables ICU‐1 (3 cages) (1)	AMX, AMC, CTX, ATM, FOX, CAZ, GM, TE
*Enterobacter cloacae*		Equine stables ICU‐2 (2 cages) (1)	AMX, AMC, FOX, CTX, CAZ, GM, SXT, TE
		Equine stables ICU‐2 (2 cages) (1)	AMX, AMC, FOX, CTX, CAZ, GM, SXT, TE
*Enterobacter cloacae*	ECL4 (2)	Residents' area (2)	AMX, AMC, FOX, CTX, MEM, SXT, TE
*Enterobacter cloacae*	ECL5	Equine stables ICU‐2 (2 cages)	AMX, FOX, CAZ, GM, SXT
*Enterobacter cloacae*	ECL6	Cat Hospitalisation room	AMX, AMC,FOX, CTX, CAZ, GM, AN, SXT, TE
*Enterobacter cloacae*	ECL7	ICU Hospitalisation	AMX, AMC, FOX,NA, CIP, SXT, TE
*Enterobacter cloacae*	ECL8	Large Dog Hospitalisation room	ATM, FOX, CTX
*Enterobacter cloacae*	ECL9 (3)	Equine stables ICU‐1 (3 cages) (3)	AMX, AMC, FOX, CTX, CAZ, GM, AN, NA, SXT, TE
*Enterobacter cloacae*	ECL10	Equine stables ICU‐2 (2 cages)	AMX, GM, SXT, TE
*Enterobacter cloacae*	ECL11 (2)	Equine stables ICU‐2 (2 cages) (2)	AMX, AMC, ATM, FOX, CTX, CAZ, GM, SXT, TE
*Enterobacter cloacae*	ECL12	Equine stables ICU‐2 (2 cages)	NO RESISTANCE
*Enterobacter* spp.	ET1	Residents' area	AMX, AMC, FOX, CAZ, GM, SXT, TE
*Enterobacter* spp.	ET2	Equine Examination Room 1	GM, SXT, TE
*Enterobacter* spp.	ET3	Infectious small animal Hospitalisation	AMX
*Enterobacter kobei*	EK1	Equine Operating Room 2	AMX, AMC, CTX, GM, NA, SXT, TE
*Pantoea agglomerans*	PA1	Consultation 2	NO RESISTANCE
*Pantoea agglomerans*	PA2	Large Dog Hospitalisation room	NO RESISTANCE
*Pantoea agglomerans*	PA3	Equine Recovery Room 2	NO RESISTANCE
*Pantoea agglomerans*	PA4	Large Dog Hospitalisation room	AMX
*Pantoea agglomerans*	PA5	ICU Hospitalisation	NO RESISTANCE
*Pantoea agglomerans*	PA6 (3)	Cat Hospitalisation room (1), small dog hospitalisation room (1), Large Dog Hospitalisation room (1)	NO RESISTANCE
*Pantoea* spp.	PT1	Consultation 2	AMX
*Pantoea* spp.	PT2	Large Dog Hospitalisation room	NO RESISTANCE
*Pantoea* spp.	PT3	Small dog hospitalisation room	NO RESISTANCE
*Pantoea conspicua*	PC	Equine Anaesthesia Induction Room 1	AMX, AMC, GM, SXT, TE
*Pantoea vagans*	PV	X‐ray room for equines	NO RESISTANCE
*Pantoea eucrina*	PE	Large Dog Hospitalisation room	NO RESISTANCE
*Leclercia adecarboxylata*	LA1	Cat Hospitalisation room	AMX, AMC, GM, SXT, TE
*Leclercia adecarboxylata*	LA2	Equine stables ICU‐1 (3 cages)	SXT
*Leclercia adecarboxylata*	LA3	Residents' area	AMX, AMC, FOX, CTX, CAZ, MEM, AN, NA, SXT, TE
*Leclercia adecarboxylata*	LA4	Equine stables ICU‐2 (2 cages)	AMX, ATM, FOX, GM, SXT, TE
*Leclercia adecarboxylata*	LA5	EquineRecovery Room 2	AMX, GM, NA, SXT, TE
*Escherichia coli*	EC1	Equine examination room 2	NO RESISTANCE
*Escherichia coli*	EC2	Small animal Anaesthesia Induction Room (1), Equine stables ICU‐1 (3 cages)	AMX, AMC, NA, CIP, SXT, TE
*Escherichia coli*	EC3	Residents' area	NO RESISTANCE
*Escherichia coli*	EC4	Equine stables ICU‐1 (3 cages)	NO RESISTANCE
*Escherichia coli*	EC5	Equine stables ICU‐1 (3 cages)	AMX, CTX, GM, SXT, TE
*Escherichia coli*	EC6	Equine stables ICU‐2 (2 cages)	AMX, GM, AN, SXT, TE
*Escherichia vulneris*	EV	Equine Recovery Room 1	AMX, CAZ, GM, SXT, TE
*Raoultella ornithinolytica*	RO1 (2)	Equine stables ICU‐1 (3 cages)	AMX, GM
*Raoultella terrigena*	RT1	Equine stables ICU‐2 (2 cages)	AMX, ATM, CTX, CAZ, SXT
*Raoultella terrigena*	RT2	Equine stables ICU‐2 (2 cages)	AMX, GM, NA, SXT, TE
*Raoultella* spp.	RSP	Equine stables ICU‐2 (2 cages)	AMX, GM, SXT
*Citrobacter freundii*	CF	Large Dog Hospitalisation room	NO RESISTANCE
*Pseudomonas putida*	PP1 (4)	Consultation 4 (1), ICU hospitalisation, equine operating room 1 (1)	AMX, AMC, FOX, MEM, SXT
*Pseudomonas putida*	PP2	Equine Examination Room 2	AMX, GM, SXT
*Pseudomonas oryzihabitans*	PO1	Small dog hospitalisation room	NO RESISTANCE
*Pseudomonas oryzihabitans*	PO2	X‐ray and CT room for small animals	ATM, CIP
*Pseudomonas mendocina*	PM1	Equine Anaesthesia Induction Room 1	NO RESISTANCE
*Pseudomonas* spp.	PSP1	Cat Hospitalisation room	NO RESISTANCE
*Pseudomonas* spp.	PSP2	Large Dog Hospitalisation room	NO RESISTANCE
*Pseudomonas* spp.	PSP3	Small animal recovery area	AMX, AMC, FOX
*Pseudomonas* spp.	PSP4	X‐ray and CT room form small animals	ATM, CIP
*Pseudomonas* spp.	PSP5	EquineExamination Room 2	SXT
*Pseudomonas* spp.	PSP6	Residents' area	NO RESISTANCE
*Pseudomonas* spp.	PSP7	Equine Recovery Room 2	GM, SXT
*Pseudomonas stutzeri*	PS1	Equine stables ICU‐1 (3 cages)	AMX, GM, SXT
*Pseudomonas stutzeri*	PS2	Equine stables ICU‐1 (3 cages)	AMX
*Pseudomonas stutzeri*	PS3 (2)	Residents' area	AMX, GM, SXT
*Pseudomonas stutzeri*	PS4	Equine Examination Room 2	AMX, GM, SXT
*Pseudomonas fluorescens*	PF1	Large Dog Hospitalisation room	NO RESISTANCE
*Pseudomonas alcaliphila*	PAL1	Equine Anaesthesia Induction Room 1	AMX, ATM, CAZ, SXT

Abbreviations: AMC, amoxicillin‐clavulanic acid; AMX, amoxicillin; AN, amikacin; ATM, aztreonam; CAZ, ceftazidime; CIP, ciprofloxacin; CS, colistin; CT, computed tomography; CTX, cefotaxime; FOX, cefoxitin; GM, gentamicin; ICU, intensive care unit; PM, imipenem; MEM, meropenem; NA, nalidixic acid; SXT, trimethoprim/sulfamethoxazole; TE, tetracycline; TGC, tigecycline.

## 4 Discussion

In this study, environmental samples were collected from several surfaces within a veterinary hospital where both animals and staff are regularly present. Different bacterial strains, including multidrug‐resistant species, were isolated from different locations in the Alfonso X el Sabio Veterinary Clinic Hospital. Data obtained from these isolates enabled the creation of an environmental map highlighting the distribution of Gram‐negative bacteria. The movement of personnel between these areas appears to play a key role in the dissemination of resistant bacteria within the veterinary hospital environment.

Although studies on the identification of bacterial strains are usually conducted using a single identification technique, in the present survey, bacterial identification was performed using biochemicals (Mehraban et al. 2016; Sánchez et al. 2015; Jara, Avendaño, and Navarro 2009), MALDI‐TOF (Zahornacký et al. 2022; Giacon, Siqueira, and Da Motta 2021; Ortiz‐Díez et al. 2023) and PCR (Morris and Cerceo 2020). Of the 109 Gram‐negative isolates obtained, 76.15% were identified as *Enterobacteriaceae* and 23.85% as NFGNB. This percentage differs from values reported by different authors (Mehraban et al. 2016; Zahornacký et al. 2022; Zurita, Garland, and Ryan 2023). Several species within the genera *Pseudomonas*, *Klebsiella* and *Enterobacte*r, identified in the present study as potentially resistant to antimicrobials, are the subject of both human and veterinary surveillance programs (ESKAPE pathogens) (Ecdc 2020; De Oliveira et al. 2020; Mulani et al. 2019). Notably, a high level of resistance to ceftazidime (CAZ) and cefotaxime (CTX) was observed in *Enterobacteriaceae*, which is eight‐fold higher than that in NFGNB. This significant difference suggests that *Enterobacteriaceae* may harbour specific resistance mechanisms, potentially linked to particular clones or bacterial species. *Enterobacter* represented one‐third of the isolates identified in the current study, with 28.91% corresponding to *E. cloacae* , a commensal bacterium in the gastrointestinal tract of humans and animals that is usually present in the environment. This species is of increasing importance in nosocomial infections due to its growing resistance to antibiotics (Intra et al. 2023; Annavajhala, Gomez‐Simmonds, and Uhlemann 2019). In the present study, *E. cloacae* showed high resistance to β‐lactams, tetracyclines and trimethoprim/sulfamethoxazole, with notable resistance to aminoglycosides (especially gentamicin (Annavajhala, Gomez‐Simmonds, and Uhlemann 2019)), and low resistance to carbapenems. Global surveillance data highlight the emergence of the carbapenem‐resistant *E. cloacae* complex (CREC), which is an increasing risk in hospital settings (Intra et al. 2023; Annavajhala, Gomez‐Simmonds, and Uhlemann 2019). Haga clic o pulse aquí para escribir texto.

*Pantoea* spp., environmental commensals of the order Enterobacterales, have been linked to hospital infections and urinary tract infections in pets (Mirtella et al. 2021; Mani and Nair 2021; Ruan, Qin, and Li 2022). Due to rising β‐lactam resistance, surveillance has increased (Smoglica et al. 2022; Gajdács 2019). In this study, *P. conspicua* showed 100% resistance to several antibiotics, meeting the criteria for MDR, as observed in some human medicine surveys (Jara, Avendaño, and Navarro 2009; Abdalhussen and Darweesh 2016).

The genus *Klebsiella* is included in surveillance programs because of its role in nosocomial infections, particularly *K. pneumoniae* (Ecdc 2020; Mulani et al. 2019; Wareth and Neubauer 2021; Lee et al. 2021; Brisse 2005; Dong, Li, and Lai 2022). In the present study, *K. oxytoca* , an emerging pathogen that causes nosocomial infections (Brisse 2005; Dong, Li, and Lai 2022; Singh, Cariappa, and Kaur 2016; Fenosa et al. 2009; Yang et al. 2022; Moradigaravand et al. 2017), with similar virulence to *K. pneumoniae* (Yang et al. 2022), was isolated. Only one *K. pneumoniae* strain displayed 100% resistance to amoxicillin, cefoxitin, gentamicin, trimethoprim/sulfamethoxazole and tetracycline while remaining susceptible to all other antimicrobials tested. In contrast, *K. oxytoca* was sensitive to imipenem, amikacin and colistin.

Antibiotic resistance in *Escherichia*, particularly in *E. coli* , is a growing public health concern (Jara et al. 2021; Sebola et al. 2023; Murphy et al. 2010; Sidjabat et al. 2006). In the current study, 14% of the isolates belonged to this genus, with *E. coli* being the predominant member of the group. The present study demonstrated the presence of antimicrobial‐resistant *E. coli* in a veterinary hospital environment, consistent with previous studies (Tuerena et al. 2016). Contamination of the veterinary practice environment with these bacteria raises concerns because environmental bacteria may disseminate to new locations, affect animals, particularly vulnerable ones and facilitate the spread of resistance genes among susceptible *E. coli* strains (Tuerena et al. 2016). In Spain, the resistance of *E. coli* to most tested antimicrobials increased significantly from 2001 to 2012, especially against third‐generation cephalosporins, amoxicillin and ciprofloxacin. However, the resistance levels have shown some stabilisation since 2016 (European Centre for Disease Prevention and Control 2022). The current study identified three different resistance profiles: one showing 100% sensitivity, another demonstrating a low resistance profile (≤ 20%) and a third displaying a medium/high resistance profile (≤ 50%). The results obtained in the present study corroborate those reported in the literature (Jara, Avendaño, and Navarro 2009; Sanchez et al. 2002). Additionally, one strain that we identified as *Escherichia vulneris* was classified as MDR, unlike other studies where *E. vulneris* infections showed susceptibility to all β‐lactams, fluoroquinolones, trimethoprim‐sulfamethoxazole and aminoglycosides (Starnes, Soewarna, and Hollingshead 2022).

NFGNBs constituted 23.85% of the isolates identified in the current study, with *Pseudomonas* accounting for 96.5%. Among these, *Pseudomonas fluorescens* , an environmental microorganism, exhibits intrinsic antibiotic resistance and poses opportunistic pathogenic threats, particularly through its ability to form biofilms in clinical settings (Benito et al. 2012; Iseppi et al. 2020). In this study, *P. fluorescens* was susceptible to all tested antibiotics, despite reports of resistance in previous studies (Silverio et al. 2022). Consistent with earlier findings, *P. putida* exhibited in this study resistance to β‐lactams, macrolides and carbapenems, while remaining susceptible to aminoglycosides and fluoroquinolones (Kim et al. 2012; Fanelli, Caputo, and Quintieri 2021). This species also showed resistance to aztreonam, as observed in *P. mendocina* , *P. alcaliphila* , *P. stutzeri* and *P. oryzihabitans* , suggesting a shared resistance mechanism (Laborda et al. 2022; Elbehiry et al. 2022). *P. aeruginosa* , a significant nosocomial pathogen with MDR potential, was not detected in this study.

In addition to ESKAPE pathogens, other opportunistic bacteria capable of causing infections in immunocompromised individuals have been identified, like *Raoultella* spp., *Raoultella ornithinolytica* and *Raoultella terrigena* , recently isolated from dogs and cats with urinary tract infection (Smoglica et al. 2022). Despite their similarity to *Klebsiella* spp., the pathogenic potential of *Raoultella* species remains uncertain (Hajjar et al. 2020; Hong et al. 2021; Castillo‐Macías et al. 2018; Drancourt et al. 2001; Appel et al. 2021; Izard, Ferragut, and Favini 1981). A broader resistance profile than that reported in earlier studies was observed in this study for *Raoultella terrígena* (Shaikh and Morgan 2011).

Five *Leclercia adecarboxylata* isolates were identified in the current study. While previous research has shown this species to be susceptible to most antibiotics used against *Enterobacteriaceae* (Zayet et al. 2021; Stock, Burak, and Wiedemann 2004), our findings revealed significant resistance levels.

*Citrobacter* species are known to cause a broad spectrum of multidrug‐resistant infections in humans and although rarely reported in veterinary medicine, they pose a potential risk in hospital settings due to their capacity for nosocomial dissemination (Poonam et al. 2019; Harada et al. 2019). In this study, *C. freundii* showed 100% susceptibility to all antibiotics tested.

One *Stenotrophomonas maltophilia* isolate was identified in the present study. This species, commonly associated with medical devices (Albini et al. 2009; Majumdar et al. 2022; Mojica et al. 2022), is resistant to a wide range of antibiotics, including β‐lactams and carbapenems, in both human and veterinary medicine (Albini et al. 2009; Majumdar et al. 2022; Mojica et al. 2022).

This study also highlighted the presence of identical bacterial pulse types across different hospital areas, suggesting potential cross‐contamination. Although there is no direct contact between animals in different rooms, the frequent movement of veterinary staff, assistants and students rotating between hospital areas likely contributed to the transmission of bacterial clones, such as *P. putida* and *E. coli* . Considering the significance of inanimate surfaces in the occurrence of nosocomial infections, transmission can persist among different hospital compartments over extended periods (Jabłońska‐Trypuć et al. 2022). Consequently, the prevalence, distribution and antimicrobial susceptibility of species obtained from environmental samples have been increasingly investigated in human and veterinary hospitals (Mehraban et al. 2016; Sánchez et al. 2015; Jara, Avendaño, and Navarro 2009; World Health Organization 2022; De Oliveira et al. 2020; Mulani et al. 2019; Lee et al. 2021) by sampling different surfaces (Otter, Yezli, and French 2011; Simmonds‐Cavanagh 2022; Zahornacký et al. 2022; Giacon, Siqueira, and Da Motta 2021; Sebola et al. 2023; Sfaciotte et al. 2021).

Finally, bacterial isolates were found on surfaces, including cages, computer keyboards, countertops, display cases, stretchers, instruments and examination tables. Fomites are a source of nosocomial infection transmission, facilitating the spread of bacteria among animals, the environment and personnel. Bacterial contamination associated with nosocomial infections has been reported in clippers, surgical scrubs, electronic devices, stethoscopes and weight scales (Zurita, Garland, and Ryan 2023; Su et al. 2021). Suboptimal infection control measures may facilitate the dissemination of resistant bacteria across hospital zones. Such dissemination raises concerns about potential infections in both human and animal populations upon contact with contaminated surfaces.

The results of this study highlight the critical role that human movement and inanimate surfaces can play in sustaining the transmission of multidrug‐resistant bacteria in veterinary hospital environments. The relative inadequacy of preventive measures within veterinary hospitals, often attributed to a lack of specialised training compared with that for human medical facilities, presents an avenue for further research and advancement. Studies on human medical hospitals have shown that limiting the movement of doctors between departments reduces the epidemic proportion of nosocomial infections (Sebola et al. 2023). Therefore, restricting human activities in veterinary hospitals and enforcing staff hygiene standards can limit the spread of hospital‐acquired infections. Following the findings of this study, several infection control measures were implemented at the hospital to limit cross‐contamination and reduce the spread of multidrug‐resistant bacteria. These actions included physical barriers to separate small animal and equine areas, the installation of disinfectant foot‐baths and stricter protocols to restrict personnel movement between these areas. Additionally, routine surface disinfection was reinforced using chlorine‐based compounds, handwashing frequency was increased and staff were prohibited from wearing long sleeves, watches, rings or other items that could accumulate pathogens. While these interventions initially enhanced the level of environmental hygiene, we observed that maintaining consistent adherence to these protocols over time proved challenging, with compliance in handwashing and routine disinfection often declining. This underscores the need for continuous evaluation and reinforcement of infection control practices to ensure sustained effectiveness. Further studies are essential to quantitatively assess the long‐term impact of these interventions on environmental contamination in veterinary hospital settings.

Our study highlights the importance of infection control strategies in veterinary hospitals and emphasises the need for continuous research to monitor the prevalence of resistant strains and mitigate the risk of cross‐transmission. Moreover, addressing antimicrobial resistance from a global perspective is crucial, as it provides a broader understanding of resistance trends and challenges. This approach is especially important given the low number of isolates in our study. Finally, it is important to note that this study has some additional limitations. While it sampled hospital surfaces, it did not include personnel or animals admitted simultaneously, making it challenging to accurately determine the source of contamination. In addition, the surfaces were not resampled after a time delay, leaving uncertainty regarding whether standard cleaning protocols effectively eliminated the initially detected bacteria or if additional measures were required to address potential bacterial contamination, including pathogens. Additionally, this study did not investigate the presence of plasmid‐mediated resistance genes, which are known to facilitate the rapid dissemination of antimicrobial resistance within bacterial populations, particularly among Enterobacteriaceae. Future studies could benefit from examining plasmid profiles to better understand potential resistance transmission mechanisms in veterinary hospital environments. On the other hand, surface sampling is less commonly recommended for environmental screening in human hospitals due to its inability to monitor airborne pathogens continuously. However, it remains valuable in veterinary settings where animals are in closer contact with surfaces. Recent studies indicate that surface sampling can effectively identify microbial contamination in veterinary environments (Harper et al. 2013; Scarpellini et al. 2024), although there is a need to incorporate air sampling techniques and establish specific standards for veterinary hospitals. Finally, this study applied CLSI human guidelines to evaluate antibiotic susceptibility in the isolates. While differences between human and animal breakpoints could potentially introduce interpretive discrepancies, most antibiotics tested exhibited comparable breakpoints across human and veterinary standards, with minor variations observed. Additionally, many antibiotics used in this study were not covered by the veterinary standards, further justifying the use of human guidelines.

## 5 Conclusions

This study describes the prevalence and resistance patterns of Gram‐negative bacterial species in a veterinary hospital environment, highlighting significant antimicrobial resistance issues. The findings reveal a substantial presence of multidrug‐resistant (MDR) *Enterobacteriaceae* and non‐fermenting Gram‐negative bacteria (NFGNB), emphasising potential cross‐transmission risks within the hospital. Notably, high resistance levels were observed in *Enterobacter cloacae* , *Pantoea conspicua* and *Klebsiella oxytoca* , while *Escherichia coli* exhibited varied resistance profiles. The study also identified identical bacterial pulse types across different hospital areas, suggesting possible bacterial spreading facilitated by hospital staff and students. These results underscore the need for stringent infection control measures to mitigate the spread of resistant bacteria. Future studies should focus on comprehensive sampling, including personnel and animals, and evaluating the effectiveness of cleaning protocols over time to ensure the elimination of potential pathogens. Continuous surveillance and improved hygiene practices are imperative to address the ongoing threat of antimicrobial resistance in veterinary settings.

## Author Contributions

**Jesús Antonio Pérez Jiménez:** methodology, formal analysis, investigation, data curation, writing – review and editing. **Silvia Penelo Hidalgo:** validation, data curation, writing – original draft, writing – review and editing, visualization. **María‐Rosario Baquero Artigao:** conceptualization, resources, supervision, project administration, funding acquisition. **Gustavo Ortiz‐Díez:** software, formal analysis, data curation, writing – original draft, validation. **Tania Ayllón Santiago:** validation, data curation, writing – original draft, writing – review and editing, visualization, supervision.

## Conflicts of Interest

The authors declare no conflicts of interest.

## Supporting information


**Figure S1.** Location map of Alfonso X el Sabio Veterinary Clinical Hospital, including the number of isolates in each sampling zone and highlighting *E. cloacae* clones (yellow dots) resistant to cefotaxime (CTX) and ceftazidime (CAZ).

## Data Availability

The original contributions presented in the study are included in the article, further inquiries can be directed to the corresponding author.
