# Supplementary material for: Prevalence, Distribution and Antimicrobial Susceptibility of Enterobacteriaceae and Non‐Fermenting Gram‐Negative Bacilli Isolated From Environmental Samples in a Veterinary Clinical Hospital in Madrid, Spain
Source: Environ Microbiol Rep. 2024 Dec 23;16(6):e70055. doi: 10.1111/1758-2229.70055 (PMC11665279; doi:10.1111/1758-2229.70055)

Supplementary material:Figure S1. Location map of Alfonso X el Sabio Veterinary Clinical Hospital, including the number of isolates in each sampling zone and highlighting *E. cloacae* clones (yellow dots) resistant to cefotaxime (CTX) and ceftazidime (CAZ).


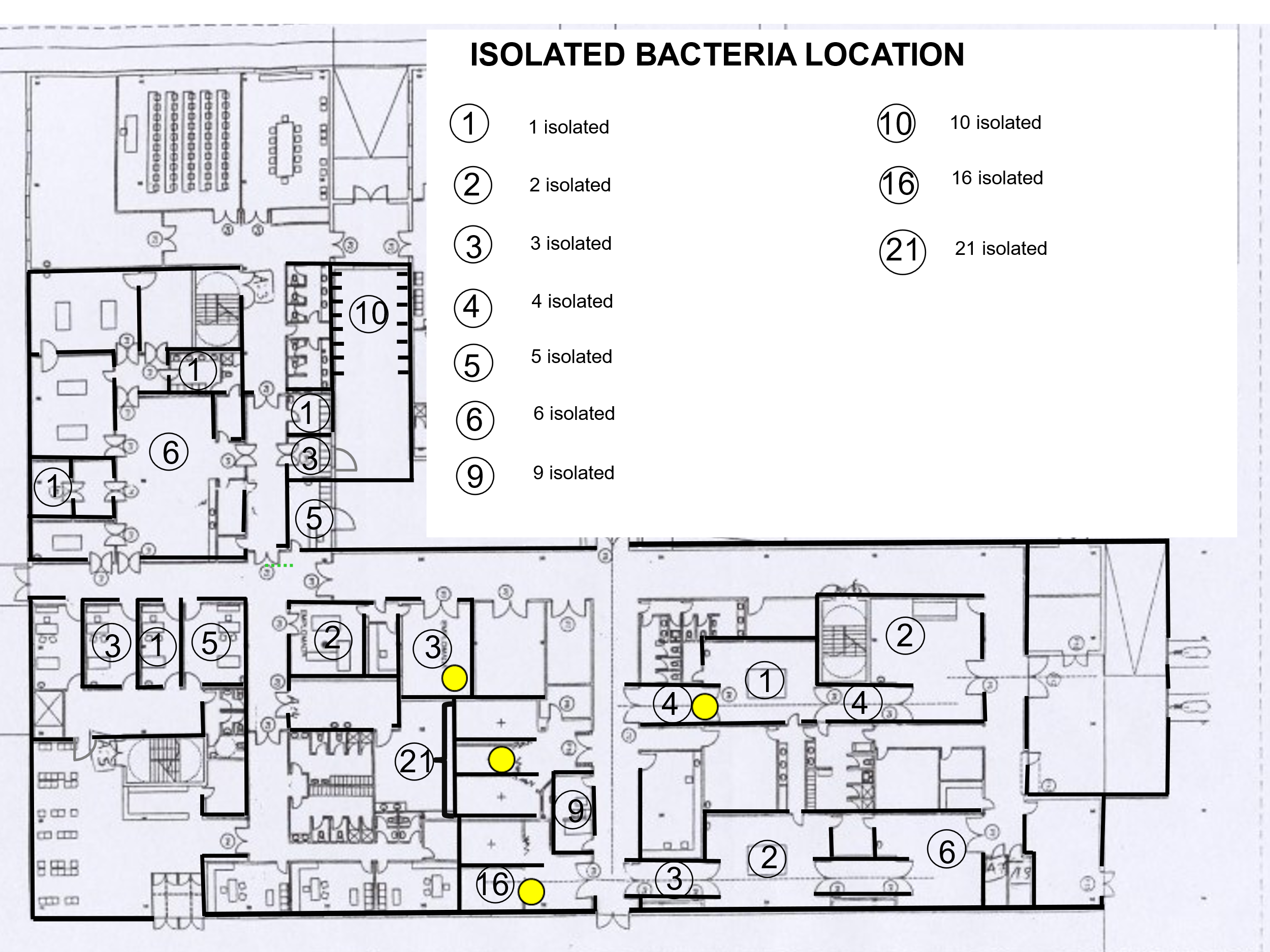

Supplement: Supplementary file 1 — Figure S1. Location map of Alfonso X el Sabio Veterinary Clinical Hospital, including the number of isolates in each sampling zone and highlighting E. cloacae clones (yellow dots) resistant to cefotaxime (CTX) and ceftazidime (CAZ). [file EMI4-16-e70055-s001.docx]
